# Intimate partner violence among young women in Ibadan, Nigeria: are there slum and non-slum differences?

**DOI:** 10.1186/s12905-023-02446-5

**Published:** 2023-05-27

**Authors:** Omowumi O. Okedare, Olufunmilayo I. Fawole

**Affiliations:** Department of Epidemiology and Medical Statistics, Faculty of Public Health, University of Ibadan, Ibadan, Nigeria

**Keywords:** IPV, Young women, Urban slum, Non-slum, Nigeria

## Abstract

This study determined the past-year prevalence of physical, sexual and psychological intimate partner violence (IPV) and associated factors among young women in urban slums and non-slums of Ibadan, Nigeria.

A cross-sectional study, using a multistage cluster sampling method was used to select 1050 ever-partnered young women aged between 18 and 24 years from the five Local Government Areas (LGAs) in Ibadan municipal. All localities were classified into slums and non-slums using the UN-Habitat 2003 criterion. Independent variables were respondents' and partners’ characteristics. Dependent variables were physical, sexual and psychological IPV. Data were analysed using descriptive statistics and binary logistic regression model (α0.05).

Prevalence of physical (31.4%, 13.4%), sexual (37.1%, 18.3%), and psychological IPV (58.6%, 31.5%) were significantly higher in the slum than non-slum communities. Multivariate analysis showed that secondary education (aOR:0.45, 95%CI: 0.21 – 0.92) reduced IPV experience while being unmarried (aOR:2.83, 95%CI: 1.28 – 6.26), partner’s alcohol use (aOR:1.97, 95%CI: 1.22 – 3.18), and partner’s relationship with other women (aOR:1.79, 95%CI: 1.10 -2.91) increased IPV experience in slum communities. In non-slum communities, having children (aOR:2.99, 95%CI: 1.05–8.51), non-consensual sexual debut (aOR: 1.88, 95%CI: 1.07–3.31) and witness of abuse in childhood (aOR:1.82: 95%CI: 1.01 – 3.28) increased experience of IPV. Acceptance of IPV and partner’s witness of abuse in childhood increased experience of IPV in both settings.

This study confirms that IPV is common among young women in Ibadan, Nigeria, but higher among women in slum communities. Findings also showed different factors associated with IPV in slum and non-slum communities. Therefore, targeted interventions for each urban stratum are recommended.

## Background

Intimate Partner Violence (IPV), any behaviour between two people in an intimate relationship that can cause physical, sexual or psychological harm, is a public health problem worldwide [1]. In addition to causing physical injury [2, 3], mental health problem [4, 5], and poor reproductive health outcome [6, 7], IPV also results in enormous social and economic loss for victims, partners, households and society [8, 9]. Likewise, IPV is a significant contributor to the global burden of disease [10].

Globally, one in every three women who have ever been in an intimate relationship has experienced at least a form of IPV in their lifetime [11, 12]. A global study by the World Health Organisation (WHO) showed that between 13 and 61% of women of reproductive age have experienced at least one form of IPV in their lifetime [13]. Compared to the global average (30%), the prevalence of IPV is lower (23.2%) in High income countries (23.2%) and higher in low/middle income countries (LMICs) (36.6%—37.7%) [11].

In both high income countries and LMICs, the prevalence of IPV is higher among adolescents and young women compared to older women [14–16]. Self-reported IPV prevalence among young women in LMICs ranged between 19% and 67.7% [14, 17–19]. The high prevalence of IPV among young people provide evidence on the early onset of violence in intimate relationships and indicates that young women are not protected from experience of IPV.

Intimate partner violence (IPV) is widespread in Sub-Saharan Africa (SSA) [11]. The cultural norms in African societies that support gender roles and acceptability of IPV contribute largely to the high level of IPV experience. Evidence show that women are more likely than men to accept IPV [18, 20, 21]. The high level of acceptance of wife beating contribute to the high prevalence of physical IPV and other forms of IPV recorded in the region. In addition, there is a culture of silence about violence in Africa, especially those that occur within marriage [22]. Similarly, IPV is seen as normal chastisement of erring wife by the husband. In Nigeria, like other developing countries, IPV is often treated as a family affair that should be settled between the couples, and not reported to law enforcement agencies [22–24]. Hence, victims often do not report and seek help, and thus, perpetrators are not prosecuted [22, 25].

Intimate partner violence is prevalent in Nigeria, because of dominant patriarchy and social norms that support male control of decision-making, family resources, economy and reproduction. Studies have shown that men and women living in households where women lack decision-making and financial autonomy were more likely to support women abuse and IPV [26, 27]. Similarly, studies have found that women in low-income communities like slums and rural areas, who are uneducated tend to endorse perpetration of IPV and experience more IPV [27].

In a qualitative study conducted in rural and urban communities of Ibadan, women attributed the causes of IPV to women’s stubborn attitude, extramarital affairs by either partner, women denying the partner sex, not obeying his instructions, disobedience, and non-submission to the partner, late food preparation, men regarding women as inferior and thus “considering women as men’s possession”, inappropriate dressing and keeping friends that the partner does not approve of were causes of IPV [28]. Women sometimes allude cause of IPV to the attitude of women and exonerate men, pointing to the existing social norms that expect women to be submissive and accept any form of maltreatment from the partner in order to keep her home. In Nigeria, many women stay in abusive relationships because of their children, societal expectation to remain married and lack of social support system [28, 29].In Nigeria, women are aware of what constitutes abuse and the different types of abuse that women experience [30]. However, many women do not consider their experience of IPV as a crime because they have been brought up to belief the man is the head of the home, and a woman must be submissive to the husband [31]. Thus, existing socio-cultural norms forbid women from speaking out about IPV experiences or leave abusive relationships. In cases, where the woman insists on leaving the abusive union, she and her family are forced to pay back the dowry [31]. Thus, family members encourage women to stay in abusive relationships and endure. This situation is worse in slums and other low-income communities because of prevalent low level of education, poverty and women subjugation.

According to the ecological framework by Heise [32], no one factor can sufficiently predict IPV. Qualitative and quantitative research have established that multiple and overlapping risk factors increase the chance of IPV occurrence. Public health also recognizes that IPV occurs in clusters within some regions and among localities [33, 34]. Based on the power theory [35], some of the factors that increase vulnerability to IPV include – low level of education, low economic status, poverty, alcoholism, experience at sexual debut, adherence to traditional norms and prominent gender roles [18, 19, 36–38]. Other factors that contribute to increased IPV experience among young women are early sexual initiation, low or no earning power, limited access and possession of resources [36, 39, 40], and wide age gap between partners [24].

In Nigeria, many young people reside in the urban areas (43%), with larger proportion (63%) residing in the urban slums, yet, only little information on IPV in urban slums in Nigeria exist. A few studies have attempted to compare IPV in urban and rural areas [41, 42], however, no documented studies have compared IPV experience in urban slums and urban non-slums in Nigeria. Comparison of IPV experience in urban slums and non-slums is important because prior IPV studies conducted in urban areas provide a summary of IPV prevalence and do not consider the different socio-economic strata within the urban area. However, using an urban data summary may mask the true prevalence in the slum communities. Similarly, different strata in the urban area will require different interventions to reduce IPV experience. We hypothesize that there is no significant difference in the experience of IPV in slum and non-slum communities of Ibadan, Nigeria. Thus, this study determined the prevalence and correlates of IPV among young women in urban slums and non-slums of Ibadan, Nigeria.

## Method

### Study settings and study design

The study was conducted in the five Local Government Areas (LGAs) of Ibadan municipal, Oyo State. Ibadan is the capital of Oyo State and the third largest metropolitan city in Nigeria, after Lagos and Kano. The estimated growth rate of 2.54% gives a population of 3,552,000 (2020) from the 2,567,000 in 2006 population census [43]. There is slight female preponderance (51%) in the population, while adolescents and young women are 20% of the total female population. Ibadan has the second largest urban concentration in South-West Nigeria. The town has more indigenous population than other towns is South-Western states. Although Ibadan is an urban city, it has pockets of traditional settlements popularly called “agbo-ile” (otherwise known as slums) in its innermost part and other middle to high income communities in its surrounding.

This study was a cross-sectional household survey, conducted among ever-partnered young women aged between 18 and 24 years. A multistage cluster sample design was adopted. All the wards in selected LGAs were stratified into slums and non-slums using pre-selected criteria [44, 45]. From the list generated, one ward each was randomly selected in the slum and non-slum per LGA. The names of communities/localities in each ward were obtained and one community was randomly selected each in the slum and non-slum. For each selected community, boundaries of selected EAs were identified and mapped by the community mobilizers. These are local resident in the community who have been involved in other health projects in the community, and are respectable in the community. The mobilizers identified all households and young women within the selected EAs.

### Study population

For the current study, all young women in selected communities have equal chance of being recruited into the study. Young women (18 – 24 years) were eligible for inclusion in the study if they have ever been or currently in a relationship, resident in selected communities for at least six months prior the study. In households with more than one eligible young women, one person was randomly selected. Young women were excluded if they were sick or not available on the day of the interview.

#### Sample size determination

Sample size was calculated using sample size method for two independent proportions. The minimum number of young women required in each study group was determined to be 458, assuming a type-1 error of 5%, 90% power, 5% non-response rate and design effect of 2.0. An estimate of the prevalence of IPV in slum and non-slum communities was obtained from a Bangladesh study [46] which reported prevalence of 41% in slum communities and 26% in non-slum communities. A total of 1050 respondents (526 slum and 524 non-slum communities) were interviewed.

#### Sampling technique

A multistage sampling technique was used. The levels of selection were ward, community and household. The wards in each LGA were stratified into slum and non-slums using selected criteria [44]. One slum ward and one non-slum ward were randomly selected from the list generated. A list of all the communities in selected wards was obtained. Ten communities each from the slum and non-slum were randomly selected. All households in the selected communities were visited, and only one young woman aged between 18 and 24 years was interviewed per household. Young women were first screened to know if they meet the eligibility criteria, and in households with more than one eligible young women, one respondent was selected by balloting.

### Instruments for data collection

Data collection tool was a semi-structured questionnaire adapted from the WHO VAW study instrument [47]. The questionnaire for this study had three sections:

- Section A- obtained information on the socio-demographic characteristics of the respondents (age, length of stay in the community, religion, ethnicity, level of education, employment status, monthly income, marital status, type of marriage, duration of relationship, number of children, number of other siblings, parent’s demographics) and her partner (age, religion, ethnicity, level of education, employment status, monthly income, alcohol use, relationship with other women).
- Section B- elicited information on attitude to IPV. This section obtained information on gender norms/roles, normalization of violence, acceptance of IPV, and social norms concerning violence.
- Section C- assessed experience of IPV (physical, controlling behaviour, psychological, economic, and sexual) and other experiences (age at sexual debut, experience at sexual debut, and witness of abuse as a child).

### Measures

#### Dependent variable

The outcome variable was past year experience of physical, sexual and psychological violence by young women. Past year prevalence of IPV was defined as the proportion of ever-partnered young women who reported to have experienced one or more acts of IPV by an intimate partner in the past one year prior to the interview. A young woman has experienced physical IPV if she had been slapped, pushed, hit with the fist, punched or kicked, choked on purpose, or partner has threatened to use or actually used a weapon against her. Sexual violence occurred if a young woman was physically forced to have sexual intercourse when she did not want or had sexual intercourse because of fear of the partner. A young woman has experienced psychological IPV if she has been insulted, belittled/humiliated, scared or intimidated by partner on purpose, threatened or had her property or valuables destroyed.

#### Independent variables

The independent variables for this study were in three categories; socio-demographic characteristics of respondents; socio-demographic characteristics of the partners; attitude towards IPV. Socio-demographic characteristics of respondents included, age (grouped as 18–19, and 20–24); ethnicity (Yoruba, Igbo, Others); religion (Christianity or Islamic); education (below secondary, secondary and tertiary); marital status (never married, married, cohabiting, separated/divorced); employment status (yes/no); income (< N20,000, N20,000 – N30,000; > N30,000); presence of children (yes/no); number of children (1, > 1) witness of abuse as a child (yes/no); age at sexual debut; experience at sexual debut (consensual, non-consensual).

Socio-demographic characteristics of the partners included: Age (≤ 24 years, > 24 years), religion (Christianity or Islamic); education (below secondary, secondary and tertiary); employment status (yes/no), ethnicity (Yoruba, Igbo, Others); income level (< N20,000, N20,000 – N30,000; > N30,000); alcohol use (yes/no); relationship with other women (yes/no); witness of abuse as a child (yes/no).

This study adopted a composite measure on acceptance of IPV using six questions. A binary variable, acceptability of IPV was generated by asking respondents whether a husband is justified to beat his wife if she; (1) neglects the children (2) argues with her husband (3) refuses to have sex with the husband (4) Goes out without telling him (5) Burns the food (6) Is suspected to have been unfaithful. The responses were: no = 0 and yes = 1. Respondents who answer “no” to all of the questions were coded “0”, and those who answered “yes” to one or more questions were coded “1”.

### Data collection techniques

The instrument for data collection was developed in English language and translated to Yoruba language. Questionnaires were interviewer-administered using the Computer Assisted Personal Interview (CAPI), a face-to-face data collection method that utilize portable devices- android phone, tablets or computer. The method of data collection did not allow for missing data and non-response. Data collectors were unable to proceed to the next question if any question was unanswered. The questionnaire took between 30 and 50 min to answer. Non-response rate was very low (*n* = 26/2.5%), majority of which were from the non-slum communities (*n* = 18/1.7%). In the non-slum the reason given was the duration of the interview, while in the slum it was due to the presence of the partners or family members. Prior to commencement of data collection, six research assistants with experience in conducting IPV research were recruited and trained on the use of CAPI and ethical conduct of IPV research.

### Data management and analysis

Statistical analyses were performed using STATA 16. Descriptive statistics using frequency and percentage were used to summarise socio-demographic characteristics of respondents and partners, acceptance of IPV, and prevalence of IPV.

Bivariate analysis using the Chi-square test was used to test for association between IPV (physical, sexual, and psychological) and independent variables (respondents’ and partners’ socio-demographic characteristics, acceptance of IPV, experience at sexual debut, and witness of abuse in childhood). Binary logistic regression using adjusted odds ratio was calculated to determine the strength of association between the independent variables an IPV. Level of statistical significance was set at α_0.05_.

### Ethical considerations

Ethical approval was obtained from the University of Ibadan/University College Hospital (UI/UCH) Joint ethics review committee. Verbal informed consent was obtained from each respondent after explaining the purpose of the research. The study adhered to the WHO ethical consideration recommended for IPV research [48]. Confidentiality of each participant was ensured by asking questions individually and not in group, and interviews were conducted in privacy for each respondent. Topic of discussion was changed to menstrual hygiene when there was any interruption. Young women were referred to organisations that support victims of domestic abuse in Ibadan. The researcher identified NGOs that support victims of domestic abuse, the support provided include: legal, shelter, empowerment and support group.

## Results

### Socio-demographic characteristics of respondents

A total of 1,050 young women participated in the study, 524 from non-slum and 526 from the slum. Table 1 shows the socio-demographic characteristics of respondents in the non-slums and slums. Higher rate of no education was found among the slum women (10.6%), while higher educational attainment was more common among non-slum women (30.9%). Majority of slum women were Muslims (61.2%) in contrast to 32.8% Muslim women in the non-slum. There were more married women in the slum (26.2%) than in the non-slum (9.4%). More young women in the slum (45.2%) had children, compared to 15.6% in the non-slum.

**Table 1:** Socio-demographic characteristics of respondents

Socio-demographic characteristics	Slum (*N* = 526) Frequency (%)	Non-slum (*N* = 524) Frequency (%)
**Age (years)**	**21.02 ± 2.08**	**21.01 ± 2.06**
18 – 19	154 (29.3)	151 (28.8)
20—24	372 (70.7)	373 (71.2)
**Level of education**		
None/Primary	56 (10.6)	21 (4.0)
Secondary	431 (81.9)	341 (65.1)
Tertiary	39 (7.4)	162 (30.9)
**Religion**		
Christianity	204 (38.8)	352 (67.2)
Islamic	322 (61.2)	172 (32.8)
**Ethnicity**		
Yoruba	518 (98.5)	445 (84.9)
Igbo	4 (0.8)	42 (8.0)
Others	4 (0.8)	37 (7.1)
**Are you working?**		
No	337 (64.1)	347 (66.2)
Yes	189 (35.9)	177 (33.8)
**Level of income**		
< N20,000	115 (60.8)	94 (53.1)
N20,000 – N30,000	51 (27.0)	67 (37.9)
Above N30,000	23 (12.2)	16 (9.0)
**Marital status**		
Never married, but was/is in a relationship	285 (54.2)	451 (86.0)
Cohabiting	96 (18.3)	23 (4.4)
Married	138 (26.2)	49 (9.4)
Separated/divorced	7 (1.3)	1 (0.2)
**Do you have children?**		
No	288 (54.8)	442 (84.4)
Yes	238 (45.2)	82 (15.6)
**Number of children**		
1	177 (74.4)	60 (73.2)
2 and more	61 (25.6)	22 (26.8)
**Type of family background**		
Monogamous	263 (50.0)	350 (66.8)
Polygamous	263 (50.0)	174 (33.2)
**Ever had sex**		
No	86 (16.3)	177 (33.8)
Yes	440 (83.7)	347 (66.2)
**Experience at sexual debut**		
Consensual	213 (40.5)	178 (51.3)
Non-consensual	227 (51.6)	169 (48.7)
**Witness of abuse as a child**		
No	355 (67.5)	380 (72.5)
Yes	171 (32.5)	144 (27.5)
**Acceptance of IPV**	292 (55.5)	122 (23.3)

On the experience of respondents at sexual debut, more than half (51.6%) of respondents in the slum reported non-consensual sexual debut, in contrast to 48.7% of respondents in the non-slum. About one-third of slum women (32.5%) reported to have witnessed abuse in childhood compared to 21.3% of non-slum women. Majority of young women in the slum (55.5%) justified perpetration of IPV, in contrast to 23.3% in the non-slum.

### Socio-demographic characteristics of partners

The socio-demographic characteristics of respondents’ partners both in the non-slum and slum are presented in Table 2. About one-third of partners in the slum (32.7%) were aged 24 years or less in the slum, compared to 41.6% in the non-slum. In terms of educational attainment, majority of partners in the slum had secondary education (74.0%), while majority of partners in the non-slum (58.6%) had tertiary education. There were more Muslim partner in the slum (67.1%) in contrast to the non-slum (35.6%). About one-third of partners in the slums (32.1%) reportedly had relationship with other women compared to 18.3% in the non-slums. Almost the same proportion of partners in the slum (42.6%) and non-slum (41.0%) reportedly drank alcohol. More partners in the slum (20.3%) reportedly witnessed abuse as a child compared to 15.6% in the non-slum.

**Table 2:** Socio-demographic characteristics of respondents’ partner

Socio-demographic characteristics	Slum (*N* = 526) Frequency (%)	Non-slum (*N* = 524) Frequency (%)
**Age (years)**	**26.67 ± 4.74**	**25.67 ± 4.14**
≤ 24	172 (32.7)	218 (41.6)
> 24	354 (67.3)	306 (58.4)
**Level of education**		
None/Primary	20 (3.8)	1 (0.2)
Secondary	389 (74.0)	216 (41.2)
Tertiary	117 (22.2)	307 (58.6)
**Religion**		
Christianity	173 (32.9)	340 (64.9)
Islamic	353 (67.1)	184 (35.1)
**Ethnicity**		
Yoruba	512 (97.3)	445 (84.9)
Igbo	8 (1.5)	46 (8.8)
Others	6 (1.1)	33 (6.3)
**Working status**		
No	110 (20.9)	156 (29.8)
Yes	416 (79.1)	368 (70.2)
**Monthly income**		
< N20,000	87 (20.9)	48 (13.0)
N20,000 – N30,000	156 (37.5)	92 (25.0)
Above N30,000	173 (41.6)	228 (62.0)
**Relationship with other women**		
No	357 (67.9)	428 (81.7)
Yes	169 (32.1)	96 (18.3)
**Alcohol use**		
No	302 (57.4)	309 (59.0)
Yes	224 (42.6)	215 (41.0)
**Witness of abuse as a child**		
No	419 (79.7)	442 (84.4)
Yes	107 (20.3)	82 (15.6)

### Prevalence of IPV in non-slum and slum

The prevalence of physical, sexual, and psychological are presented in Table 3. Majority (55.0%) of young women interviewed had experienced psychological IPV, 22.7% experienced sexual IPV, and 22.4% experienced physical IPV.

**Table 3:** Prevalence of IPV in non-slum and slum

Type of IPV	Overall (*n* = 1050) Frequency (%)	Slum (*n* = 526) Frequency (%)	Non-slum (*n* = 524) Frequency (%)	Test statistics (χ^2^)	*p*-value
Physical	235 (22.4)	165 (31.4)	70 (13.4)	49.013	0.000
Sexual	291 (27.7)	195 (37.1)	96 (18.3)	46.073	0.000
Psychological	578 (55.0)	308 (58.6)	270 (51.5)	5.240	0.022

The prevalence of different types of IPV was significantly higher in the slum than non-slum (*p* < 0.05). Psychological IPV was the most prevalent type of IPV in both slum (58.6%) and non-slum (51.5%), while physical IPV was the least prevalent type of IPV in the non-slum (13.4%) and slum (31.4%).

### Predictors of past-year experience of IPV

Table 4 shows respondents and partner characteristics that predict physical IPV in both non-slum and slum. In the non-slum, women who had children were more than twice likely to experience physical IPV (OR: 2.99, 95%CI = 1.05 – 8.51). The odds of experiencing physical IPV was higher among women who accepted IPV (OR: 1.96, 95% CI = 1.07 – 3.57) and witnessed abuse as a child (OR: 1.82, 95% CI = 1.01 – 3.28).

**Table 4:** Predictors of physical IPV and respondents/partners characteristics

Variables	Slum AOR (95% CI)	Non-slum AOR (95% CI)
**Level of education**		
None/Primary	Ref	Ref
Secondary	**0.45 (0.21 – 0.92)**	0.41 (0.14 – 1.23)
Tertiary	0.55 (0.16—1.90)	0.57 (0.16 – 2.00)
**Working status**		
No	Ref	
Yes	1.32 (0.80 – 2.17)	
**Marital status**		
Never married	**2.83 (1.28 – 6.26)**	1.36 (0.36 – 5.15)
Cohabiting	1.30 (0.60 – 2.84)	1.33 (0.43 – 4.20)
Married	Ref	Ref
Separated/divorced	1	1
**Presence of children**		
No	Ref	Ref
Yes	1.66 ((0.84 – 3.29)	**2.99 (1.05 – 8.51)**
**Family background**		
Monogamous		Ref
Polygamous		1.55 (0.87 – 2.77)
**Acceptance of IPV**		
No		Ref
Yes	**2.01 (1.21 – 3.32)**	**1.96 (1.07 – 3.57)**
**Witness of abuse**		
No		Ref
Yes		**1.82 (1.01 – 3.28)**
**Partner’s age**		
≤ 24		Ref
> 24	0.60 (0.32 – 1.15)	1.13 (0.58 – 2.19)
**Partner’s level of education**		
None/Primary	Ref	Ref
Secondary	0.81 (0.26 – 2.53)	1.10 (0.57 – 2.10)
Tertiary	0.70 (0.19 – 2.52)	1
**Partner’s alcohol use**		
No	Ref	Ref
Yes	**1.97 (1.22 – 3.18)**	1.65 (0.94 – 2.91)
**Partner’s relationship with other women**		
No		Ref
Yes		1.60 (0.84 – 3.06)

In the slum, having secondary education reduces the odds of experiencing physical IPV (OR: 0.45, 95% CI = 0.21—0.92). However, the odds of experiencing physical IPV was higher among women who were never married (OR: 2.83, 95% CI = 1.28 – 6.26), accepted IPV (OR: 2.01, 95% CI = 1.21 – 3.32), whose mother had tertiary education (OR: 3.68, 95% CI = 1.35 – 10.07), whose parents were separated/widowed (OR: 1.59, 95% CI = 0.99 – 2.54), and whose parents drank alcohol (OR: 1.97, 95% CI = 1.22 – 3.18).

While partners’ age above 24 years was a risk factor for experiencing physical IPV in the non-slum (OR: 1.13, 95% CI = 0.59 – 2.19), it was protective in the slum (OR: 0.60, 95% CI = 0.32 – 1.15).

The predictors of sexual IPV in both non-slum and slum are presented in Table 5. In the non-slum, risk factors for sexual IPV were acceptance of IPV (OR: 2.13, 95% CI = 1.14 – 3.96), secondary education of mother (OR: 2.26, 95% CI = 1.06 – 4.82), and partners’ witness of abuse (OR: 2.31, 95% CI = 1.16 – 4.58).

**Table 5:** Predictors of sexual IPV and respondents/partners characteristics

Variables	Slum AOR (95% CI)	Non-slum AOR (95% CI)
**Age group**		
18 – 19	Ref	Ref
20—24	0.60 (0.30 – 1.21)	1.60 (0.69 – 4.10)
**Level of education**		
None/Primary	Ref	
Secondary	0.66 (0.31 – 1.39)	
Tertiary	0.78 (0.22 – 2.76)	
**Religion**		
Christianity		Ref
Islamic		1.10 (0.53 – 2.28)
**Working status**		
No	Ref	Ref
Yes	1.10 (0.68 – 1.79)	1.47 (0.82 – 2.63)
**Marital status**		
Never married	1.12 (0.52 – 2.41)	1.26 (0.35 – 4.55)
Cohabiting	0.96 (0.46 – 2.00)	0.99 (0.34 – 2.93)
Married	Ref	Ref
Separated/divorced	0.48 (0.08 – 2.93)	1
**Presence of children**		
No	Ref	Ref
Yes	**2.63 (1.37 – 5.06)**	1.85 (0.71 – 4.86)
**Acceptance of IPV**		
No	Ref	Ref
Yes	**1.77 (1.09 – 2.86)**	**2.13 (1.14 – 3.96)**
**Experience at sexual debut**		
Consensual		Ref
Non-consensual		1.88 (1.07 – 3.31)
**Partner’s age**		
≤ 24	Ref	Ref
> 24	1.00 (0.51 – 1.97)	1.84 (0.80 – 4.22)
**Partner’s level of education**		
None/Primary	Ref	Ref
Secondary	0.46 (0.14 – 1.52)	1.81 (0.98 – 3.32)
Tertiary	0.64 (0.17 – 2.42)	1
**Partner’s religion**		
Christianity		Ref
Islamic		1.22 (0.59 – 2.49)
**Partner’s employment status**		
No		Ref
Yes	1	1.00 (0.43 – 2.32)
**Partners monthly income**		
< N20,000	Ref	
N20,000 – N30,000	0.73 (0.39 – 1.37)	
> N30,000	0.59 (0.31 – 1.11)	
**Partners’ alcohol use**		
No	Ref	Ref
Yes	**1.67 (1.05 – 2.66**)	1.63 (0.92 – 2.89)
**Partner’s relationship with other women**		
No	Ref	Ref
Yes	**1.79 (1.10 – 2.91)**	1.37 (0.70 – 2.65)
**Partner’s witness of abuse**		
No	Ref	Ref
Yes	**1.85 (1.05 – 3.25)**	**2.31 (1.16 – 4.58)**

Risk factors for sexual IPV in the slum were having children (OR: 2.63, 95% CI = 1.37 – 5.06), acceptance of IPV (OR: 1.77, 95% CI = 1.09 – 2.86), having more than three siblings (OR: 1.76, 95% CI = 1.05 – 2.95), ever having sex (OR: 12.38, 95% CI = 2.60 – 58.94), partners’ alcohol use (OR: 1.67, 95% CI = 3.05 – 2.66), partner’s relationship with other women (OR: 1.79, 95% CI = 1.10 – 2.91), and partners’ witness of abuse (OR: 1.85, 95% CI = 1.05 – 3.25).

Table 6 presents the predictors of psychological IPV in both non-slum and slum. In the non-slum, odds of psychological IPV was higher among whose father had secondary education (OR: 3.43, 95% CI = 1.30—9.05), who experienced non-consensual sexual debut (OR: 2.77, 95% CI = 1.22 – 6.27), and whose partners witnessed abuse (OR: 4.36, 95% CI = 1.34 – 14.15). Although not significant, odds of experiencing psychological abuse was higher among women who had secondary education (OR: 2.43, 95% CI = 0.29—20.54), earn above N30,000 monthly (OR: 4.27, 95% CI = 0.83 – 21.91), never married (OR: 3.99, 95% CI = 0.52 0 20.58), have more than three siblings (OR: 1.82, 95% CI = 0.75 – 4.44), and witnessed abuse (OR: 1.57, 95% CI = 0.59 – 4.06).

**Table 6:** Predictors of psychological IPV in slum and non-slum

Variables	Slum AOR (95% CI)	Non-slum AOR (95% CI)
**Level of education**		
None/Primary		Ref
Secondary		2.43 (0.29 – 20.54)
Tertiary		1.99 (0.20 – 19.35)
**Level of income**		
< N20,000	Ref	Ref
N20,000 – N30,000	**0.33 (0.15 – 0.74**)	0.57 (0.25 – 1.32)
> N30,000	0.42 (0.14 – 1.28)	4.27 (0.83 – 21.91)
**Marital status**		
Never married	2.66 (0.77 – 9.11)	3.99 (0.52 – 20.58)
Cohabiting	1.55 (0.53 – 4.57)	2.04 (0.45 – 9.23)
Married	Ref	Ref
Separated/divorced	2.48 (0.15 – 42.434)	
**Presence of children**		
No	Ref	Ref
Yes	1.72 (0.65 – 4.58)	0.77 (0.20 – 2.94)
**Family background**		
Monogamous		Ref
Polygamous		0.98 (0.41 – 2.30)
**Acceptance of IPV**		
No	Ref	Ref
Yes	2.04 (0.96 – 4.34)	0.76 (0.28 – 2.06)
**Experience at sexual debut**		
Consensual	Ref	Ref
Non-consensual	1.83 (0.90 – 3.73)	**2.77 (1.22 – 6.27)**
**Witness of abuse**		
No	Ref	Ref
Yes	2.23 (0.91 – 5.48)	1.57 (0.59 – 4.06)
**Partner’s education**		
None/Primary		
Secondary		
Tertiary		0.36 (0.14 – 0.96)
**Partner’s working status**		
No	Ref	
Yes	0.74 (0.13 – 4.12)	
**Partners alcohol use**		
No	Ref	
Yes	1.13 (0.54 – 2.36)	
**Partner’s relationship with other women**		
No	Ref	
Yes	1.68 (0.77 – 3.69)	
**Partner’s witness of abuse**		
No	Ref	Ref
s	2.04 (0.75 – 5.57)	**4.36 (1.34 – 14.15)**

In the slum, no factor significantly increased the odds of psychological IPV. However, odds was higher among women who were never married (OR: 2.66, 95% CI = 0.77 – 9.11), had children (OR: 1.77, 95% CI = 0.65 – 4.58), accepted IPV (OR: 2.04, 95% CI = 0.96 – 4.34), experienced non-consensual sexual debut (OR: 1.83, 95% CI = 0.90 – 3.73), witnessed abuse (OR: 2.23, 95% CI = 0.91 – 5.48), whose partner drank alcohol (OR: 1.13, 95% CI = 0.54 – 2.36), and whose partner had relationship with other women (OR: 1.68, 95% CI = 0.77 – 3.69).

## Discussion

To our knowledge this is the first household survey that provides baseline information on the prevalence of intimate partner violence (IPV) and associated factors among young women in slums and non-slum communities of an urban city in Nigeria and sub-Sahara Africa. This study provides evidence on the multiple risk factors that are linked to IPV among young women in Ibadan and elsewhere. The results are useful and provide proximate data for other research to build upon and make informed decision.

Our study reported an overall past-year prevalence of physical, sexual and psychological IPV as 22.4%, 27.7% and 55.0% respectively. Previous studies which have explored the prevalence of IPV among young women in Nigeria found rates ranging from 7.9% [49] to 42.3% [2, 19, 50, 51]. Our prevalence of physical and sexual violence fall within the range of estimates documented from previous studies, but prevalence of psychological IPV was higher than the 41.8% reported by Umana et al., (2014). The prevalence in this study is higher than those reported in Europe [15], Nepal [52], Bangladesh [53], sub-Saharan Africa [54], Ethiopia [55], and Nigeria [36, 56]. It has been documented that the highest occurrence of IPV happens quite early – in late adolescence and young adulthood [57]. Hence, the high prevalence reported in this study. However, a study in Dhaka slum reported higher proportion (60%) than we have in this study [58].

We found that the past-year prevalence of IPV varied by stratum, IPV was higher in the slum than non-slum. This is consistent with previous studies which reported that IPV is more likely to be reported by women in low-income settings [59, 60]. In our study, psychological IPV was the most prevalent and physical IPV was the least in both settings. This is similar to the finding of Sambisa et al., (2011) where psychological IPV was the most reported and physical IPV was the least prevalent. The high prevalence of psychological IPV confirms findings by other researchers that non-contact violence such as psychological/emotional IPV is the most prevalent type of IPV [56, 61, 62]. The result of the current study is in contrast to some other studies that reported psychological and sexual IPV as less prevalent than physical IPV [5, 14, 52, 55, 56, 63]. An explanation is that psychological IPV is often difficult to establish and the perpetrator may go unpunished. Also, physical IPV is socially acceptable in many relationships in low-income countries as a way of the man correcting his partner and showing her love, thus, it is often underreported [64, 65]. Some victims may also believe they deserve physical abuse from their partners demonstrating the generally acceptability of violence, and this may contribute to its low prevalence in this study.

The prevalence of physical IPV reported for slum in this study is consistent with that in Bangladesh [46], Egypt [66], but lower that the report from India [58].

The results of this study confirmed that many socio-demographic characteristics of young women and their partners increased the likelihood of being abused. Experience of physical IPV reduced with increasing level of education in the non-slum, while physical IPV increased with increasing level of education in the slum. The observation in the slum was not unexpected as those with lower level of education uphold the traditional norms that supports women victimization. Consistent with other studies in Tanzania [67], submission to the husband is a culturally acceptable standard and any behaviour outside of this triggers IPV. Hence, such women become subjective and continue to tolerate IPV [68].

Higher level of education was protective of IPV [46, 69], and low level of education is a risk for IPV [70, 71]. The level of women’s education improves their opportunities for employment, financial autonomy, communication skills and decision-making capacity, which consequently affect their IPV experience [54, 71].

Being married was protective of physical IPV in both slum and non-slum. Previous studies have documented that relationship status is associated with IPV, with married women experiencing the lowest risk and separated/divorced women most vulnerable [57, 61, 69, 72]. Also, cohabitation increases IPV in this study similar to previous research [54, 71]. It could be explained that women in cohabiting situation feel less empowered to challenge any form of abuse from their partners or do not understand their partners like those in formal union [54].

Consistent with previous findings [46, 51, 69, 73] acceptance of IPV increased victimization among respondents in the slum and non-slum. Women who accept a man victimizing the partner are less likely to report incidents of abuse in their union or others. Also, accepting IPV normalizes the experience of victimization and such women may not see their experiences as abnormal. Such women may accept IPV because of dependence on their partner. In a similar study among young women in urban slums in Nepal, many young women are trapped in abusive relationships because of financial dependence on their partners [68, 74].

Consistent with other studies in Kenya [15, 21], and the USA [75], exposure to violence as a child increases the risk of IPV. Witness of abuse in childhood is a strong predictor of IPV victimization in adulthood. Women who witnessed their mothers being abused mother often report higher risk of IPV compared to those who did not witness the abuse of their mothers across different settings [2, 6, 76]. A woman who witnessed the mother being abused may have grown up to believe that is the right and acceptable lifestyle in a relationship. It is also possible that she has been abused by the father during one of the moments that the mother is being abused. In such family, women would have been subjugated. Hence, such child tends to condone violence and see it as a norm [27].

Low level of partner’s education, alcohol use, and relationship with other women increases the experience of physical IPV in both non-slums and slums. Women whose partner had no formal or less than post-secondary education are more likely to experience IPV compared to women whose partner had higher education [54, 73]. It could be explained that men who have lower education status may have low self-esteem and result to using violence as the only means of conflict resolution.

Women whose partners had more than secondary education and witnessed abuse as a child in both slum and non-slum experienced more psychological abuse. Higher level of educational status translates to better opportunities and higher income. Earlier research has documented that in homes where only the man is working, there is report of more violence than where both are working [77]. This may be so because the man may be under financial pressure and continuous demand by the wife may trigger his perpetration of IPV. Inability of the man to meet the financial demand of the home may contribute to perpetration of IPV [65].

Just like in previous researches [2, 67, 78–80], alcohol use by partner significantly increased IPV experience among young women. Alcohol use impair judgement and the man may result to anger at the slightest provocation. Alcoholism may increase the financial burden on the household, which can stress the woman and lead to conflict [78, 80].

This study has some limitations. First, this study utilised a cross-sectional design, which makes it impossible to establish a causal association between IPV and selected characteristics. Nonetheless, the association reported provide insights about the role of selected characteristics on experience of IPV. Secondly, IPV was self-reported and it may likely introduce possible bias or underreporting due to social desirability effect. However, interview-administered questionnaire using CAPI helped to reduce this bias.

However, this study enhances our understanding of physical, sexual and psychological IPV among young women. It also provides data on the magnitude, pattern and associated factors in different strata in Ibadan metropolis. Consistent with the ecological framework [32], our study confirms that occurrence of IPV against young women is related to an interplay of factors. Thus, interventions to reduce IPV against young women will require strategies that take into cognizance the different levels in the ecological model.

This study is unique in that it considered important strata within the urban area i.e. slums and non-slums communities. The participants are also unique group among women of reproductive age. Studies on this distinct age group have been limited to a small sample with limited geographic coverage. Our study has been able to show that IPV is a common occurrence among young women, and not limited to either slum or non-slum alone. The high prevalence of the three types of IPV considered in this study underscores the need for public health interventions that use culturally acceptable approaches to tackling IPV. In addition, interventions must be tailored to each urban stratum for acceptability and effectiveness. In the slum, interventions should focus on community norms that promote IPV and expose children to abuse. Similarly, interventions in the slums should address substance use by partners through behavioural change programmes. In both slum and non-slums, interventions should be geared towards reducing acceptance of IPV by young women, through positive role models and education.

## Data Availability

The datasets generated during the current study are not publicly available because this manuscript is from a larger study that has not been published, but datasets are available from the corresponding author on reasonable request.
